# Nasopharyngeal Carriage of *Streptococcus pneumonia* in Pneumonia-Prone Age Groups in Semarang, Java Island, Indonesia

**DOI:** 10.1371/journal.pone.0087431

**Published:** 2014-01-31

**Authors:** Helmia Farida, Juliëtte A. Severin, M. Hussein Gasem, Monique Keuter, Hendro Wahyono, Peterhans van den Broek, Peter W. M. Hermans, Henri A. Verbrugh

**Affiliations:** 1 Department of Microbiology, Faculty of Medicine Diponegoro University - Dr. Kariadi Hospital, Semarang, Indonesia; 2 Department of Medical Microbiology and Infectious Diseases, Erasmus University Medical Centre, Rotterdam, the Netherlands; 3 Department of Internal Medicine, Dr. Kariadi Hospital - Faculty of Medicine Diponegoro University, Semarang, Indonesia; 4 Department of General Internal Medicine, Radboud University Nijmegen Medical Centre, Nijmegen, the Netherlands; 5 Department of Infectious Diseases, Leiden University Medical Centre, Leiden, the Netherlands; 6 Nijmegen Institute for Infection, Inflammation, and Immunity (N4i), Nijmegen, the Netherlands; 7 Laboratory of Paediatric Infectious Diseases, Radboud University Nijmegen Medical Centre, Nijmegen, the Netherlands; 8 Crucell - Johnson and Johnson, Leiden, the Netherlands; Facultad de Medicina, Uruguay

## Abstract

**Introduction:**

*Streptococcus pneumoniae* is a worldwide occurring pathogen Nasopharyngeal carriage of *Streptococcus pneumoniae* precedes pneumonia and other pneumococcal diseases in the community. Little is known about *S. pneumoniae* carriage in Indonesia, complicating strategies to control pneumococcal diseases. We investigated nasopharyngeal carriage of *S. pneumoniae* in Semarang, Indonesia.

**Methods:**

A population-based survey was performed in Semarang, Indonesia. Nasopharyngeal swabs and questionnaires were taken from 496 healthy young (6–60 month-old) children and 45–70 year-old adults.

**Results:**

Forty-three percent of children aged 6–60 months and 11% of adults aged 45–75 years carried *S. pneumoniae*. Determinants of carriage were being a child (OR 7.7; 95% CI = 4.5–13.0), passive smoking (OR 2.1; 95% CI = 1.3–3.4), and contact with toddler(s) at home (OR 3.0; 95% CI = 1.9–4.7). The most frequent serotypes found were 6A/B and 15B/C. The current commercially available vaccines cover <50% serotypes found in children. Twenty-four percent of *S. pneumoniae* strains were penicillin non-susceptible, and 45% were resistant to cotrimoxazol.

**Conclusions:**

The limited coverage of commercially available vaccines against the serotypes found in this population, and the high proportion of non-susceptibility to penicillin and cotrimoxazol suggest the need for region-specific information and strategies to control *S. pneumoniae*.

## Introduction

*Streptococcus pneumoniae* is a worldwide occurring pathogen [1]. Data on this species are abundantly available in developed countries, but still scarce in low-to-middle-income countries, leading to difficulties in designing national strategies to control pneumococcal diseases.

Since pneumococcal pneumonia is preceeded by nasopharyngeal colonization with *S. pneumoniae* [2], it is relevant to study the nasopharyngeal carriage pattern in humans, particularly in those at higher risk of pneumonia. Pneumococcal carriage has already been extensively studied in many parts of the world, but only few data are available from Indonesia [3], the fourth most populated country in the world. We investigated the nasopharyngeal carriage of *S. Pneumonia* in an urban area of Indonesia, to study the prevalence, risk factors, serotypes, and antimicrobial susceptibility.

## Methods

### Ethics Statement

The study was approved by The Ethical Committee of the Faculty of Medicine, Diponegoro University, Semarang. Written informed consent was given by the subjects or their caregivers.

### Subjects

A population-based survey was performed in Semarang, a city with 1.5 million residents in Central Java, among healthy children aged 6–60 months and healthy adults aged 45–70 years as described before [4]. Exclusion criteria were the presence of respiratory symptoms and antibiotic consumption within the last three days. Cluster random sampling was done from February to April 2010 to recruit subjects from all 16 districts of Semarang.

### Specimen Collection and Laboratory Testing

Nasopharyngeal swabs were obtained using rayon-tipped swabs and transported in Amies-charcoal media (COPAN, Italy). Swabs were inoculated on 5% sheep blood agar with gentamicin (5 mg/liter) and incubated at 35°C in 5% CO_2_ for 48 hours. Identification of *S. pneumoniae* was performed using the optochin test (Oxoid, Basingstoke, UK) and, in case of doubt, a DNA hybridization test (Accuprobe, Gen-Probe Inc., San Diego, CA, USA). Antimicrobial susceptibility tests were performed using disk diffusion method (Oxoid, UK) and E-test (bioMérieux, France) and interpreted according to EUCAST 2012. Serotyping of *S.pneumonia*was done with a multiplex-PCR which covers 36 serotypes [5], [6]. Control strains were included in all analyses.

Data on demography, house sanitation (crowding, smoke exposure from cigarette and mosquito coils), and water and food hygiene, were recorded using a questionnaire that was developed to identify determinants of carriage. Crowding was defined to be present when the ratio of total bedroom space to the number of family members was less than 4 m^2^ [7]. Water hygiene was defined as poor when water other than tap or bottled water was used by the family. Food hygiene was considered poor if the family consumed street food.

### Statistical Analysis

Univariate analysis was done with Chi-square or Fisher’s exact tests when appropriate, followed by backward stepwise logistic regression for variables with *P* value <0.2 using SPSS 17 (SPSS Inc, Chicago, USA). *P* value of <0.05 was considered significant.

## Results

### Subjects

Two hundred and fifty-three adults aged 45–70 years and 243 children aged 6–60 months participated in the study. The characteristics of the participants have been presented previously [4]. Crowding was common, as was exposure to smoke.

### Carriage Prevalence and Determinants

**Figure 1:**
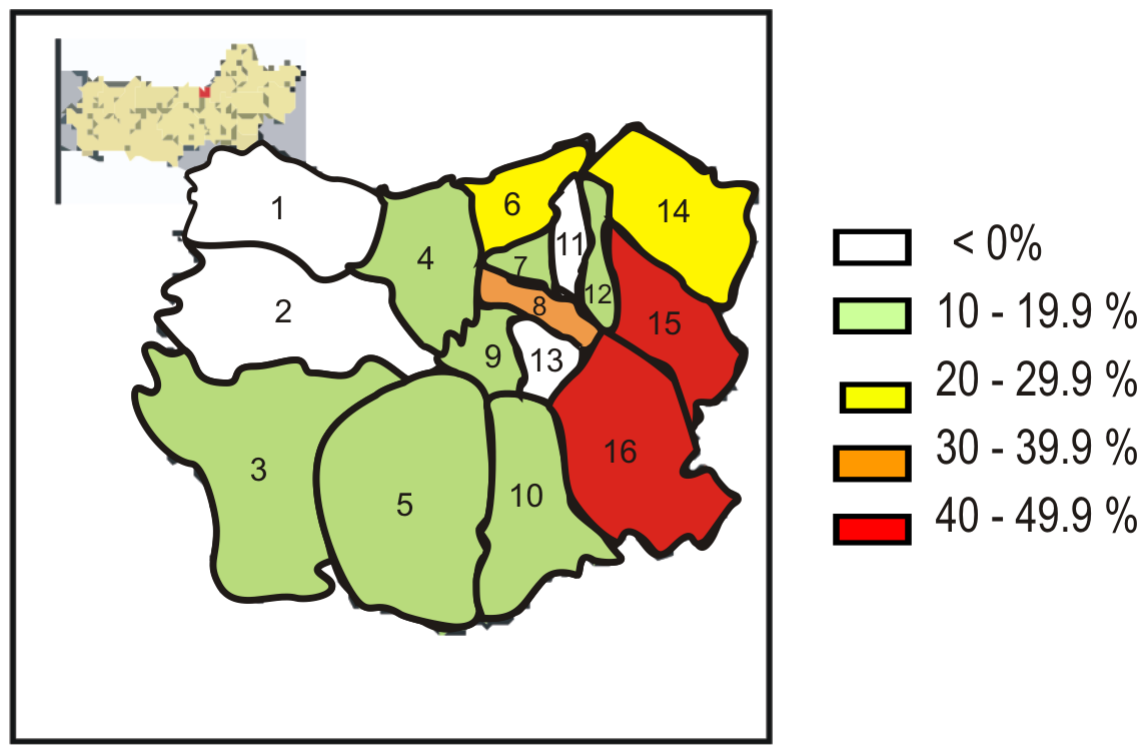
Distribution of nasopharyngeal carriage of *S. pneumoniaei* among healty population in the districts of Semarang, Indonesia.

Overall carriage of *S. pneumoniae* was 27% (95% CI: 20–32), 43% in children (95% CI: 32–50) and 11% in adults (95% CI: 5–15). The proportion carrying *S.pneumoniae* varied significantly across the districts of Semarang (*P*<0.05), and tended to be higher in the suburban and eastern parts of the city (Figure 1). Multivariate analysis showed that being a child (OR 7.7, 95% CI,4.5–13.0), passive smoking (OR 2.1, 95% CI,1.4–3.4), and contact with toddler(s) at home (OR 3.0, 95% CI, 1.9–4.7) were independent determinants of carriage.

### Antimicrobial Susceptibility and Serotypes

One hundred and forty-two strains were isolated from 133 subjects. In total, 34 (24%) strains were penicillin non-susceptible (MIC ranged 0.047–1.5), including 25 (23%) from children and 9 (29%) from adults (*P* = 0.25). Forty-five percent of the strains were resistant to cotrimoxazol, 1% to erythromycin, and 5% to tetracycline. There was no significant difference in the susceptibility pattern between isolates from children and those from adults (*P*>0.1). No strain was resistant to neither penicillin nor vancomycin.

Capsular type 6A/B was the most prevalent serotype in all age groups (19% in children and 39% in adults). The most common capsular serotypes in children, comprising 61% of strains, were 6A/B, 15B/C, 11A, 23F, 19F, 23A. Those in adults, were 6A/B, 15B/C, and 15A. These two serotype patterns differed significantly (*P* = 0.029). Other serotypes were less frequently found (Table 1). Twenty percent were un-typeable with the multiplex-PCR employed.

**Table 1:** Serotype of *S. pneumoniae* isolated from healthy people in Semarang, Indonesia.

	Children	Adults	Total	*P*
	n (%)	n (%)	n (%)	
6A/6B	21 (19)	12 (39)	33 (23)	0.029
15B/C	11 (10)	4 (13)	15(11)	
11A	11 (10)	1 (3)	12 (8)	
23F	10 (9)	1 (3)	11 (8)	
19F	9 (8)	0 (0)	9 (6)	
23A	5 (5)	0 (0)	5 (4)	
15A	2 (2)	3 (10)	5 (4)	
Others	22 (20)	2 (6)	24 (17)	
Un-typeable	20 (18)	8 (26)	28 (20)	
Total	111 (100)	31 (100)	142 (100)	

## Discussion

The carriage prevalence of *S.pneumoniae* among children in our study was comparable to those previously found among healthy children on Lombok island, Indonesia [3], and in the Netherlands [8]. However, it was lower than those in Gambia [9], Poland [10], Australia [11], Thailand [12], and higher than those reported from Iran [13] and Korea [14]. The carriage prevalence among adults in our study was 11%, wich is higher than that found in Alaska [15], but lower than that among Australian Aboriginals of the same age [11]. The prevalence differences among populations may be related to sampling or laboratory methods (i.e. nasopharyngeal swab versus throat swab, the use of selective media), to certain characteristics of the population studied (i.e. the age of the subjects, household characteristics – especially the presence of toddlers, presence of upper respiratory tract infection, vaccination status), or to seasonal variation. Our samples were taken in the rainy season, during which the incidence of respiratory tract infection, transmission of pathogens, and thus, carriage is likely to be somewhat increased.

The prevalence of *S. pneumoniae* with reduced susceptibility to penicillin and cotrimoxazole was high. The national and local guidelines for empirical antibiotics for community-acquired pneumonia in children still recommend these two antibiotics as the first choices [16], and those for meningitis recommend ampicillin for the second line [17].

The commercially available 13-valent pneumococcal conjugate vaccines (PCV13) [18], which was introduced only in 2011 in Indonesia, provides approximately 45% strain coverage for the infant population in this study, varying from 13–100% across the city districts. PCV10, introduced in 2010, provides a little bit lower coverage. However, the coverage of the PCV13 over the serotype repertoir on Lombok island in Indonesia in the past [3] was 60% and in other Southeast Asian countries, the coverage ranged from 63%–97% [19]. Our results may, thus, not be taken to reflect the serotype distribution throughout Indonesia, since the study was performed in a specific geographic location.

The PCVs have not been included in the national vaccination programs since information regarding the burden of pneumococala diseases in Indonesia is still lacking. Rather, pneumococcal vaccines have been introduced in private clinics. This vaccine is rather expensive for regular Indonesian households that it is unlikely to even now have had any significant impact on the carriage of pneumococci observed in Indonesia.

So far, studies in Indonesia failed to reveal the burden of pneumococcal diseases in Indonesia probably due to technical problems [20], [21], thus, limiting the availability of data needed to develop appropriate policies and strategies to control pneumococcal disease, which is considered as one of the most important infectious diseases, in particular for toddlers and elderly worldwide. This underscores the need for surveillance of pneumococcal disease in Indonesia using locally implementable laboratory methods.

This study provides further evidence that passive smoking is an independent determinant of *S. pneumoniae* carriage among children [10], [13]. The mechanism by which passive smoking influences the microbial ecology of the upper respiratory tract remains to be elucidated, however.

In conclusion, nasopharyngeal carriage of *S. pneumonia* was common among healthy children and adults in this urban area of Indonesia, and was determined, at least in part, by the presence of toddlers in the household and smoking habit of the adults. The low coverage of commercially available vaccine against the serotypes found among children in this urban population, and the high proportion of non-susceptibility to penicillin and co-trimoxazol suggest the need for region-specific information and strategies to control *S. pneumoniae*. A nation wide epidemiological study on pneumococcal carriage and disease throughout Indonesia would provide such data and inform public health policies.
